# ‘Changing People's Hearts’: The Lived Expertise Perspective of Communicating With Nursing Students at a Mental Health Clinical Placement

**DOI:** 10.1111/inm.13465

**Published:** 2025-01-20

**Authors:** Hannah Thompson, Christopher Patterson, Kelly Lewer, Lorna Moxham

**Affiliations:** ^1^ School of Nursing, Faculty of Science, Medicine and Health University of Wollongong Wollongong Australia

**Keywords:** clinical placement, lived experience, mental illness, nurse education, phenomenology, recovery

## Abstract

Escalating rates of mental illness emphasise the necessity for sufficient and appropriate mental health services. However, stigma and discrimination remain and can be seen through the multifaceted ways nurses communicate. Clinical placements, where nursing students engage directly with individuals experiencing mental illness, are vital for addressing these challenges by fostering empathy and reducing stigma. This study aimed to explore how individuals with lived experience of mental illness experience the communication of nursing students during their participation in the mental health nursing clinical placement, *Recovery Camp*. Using a Heideggerian phenomenological approach, five individuals with lived experience of mental illness, referred to as Experts by Experience, participated in individual semi‐structured interviews. van Kaam's psychophenomenological approach was used for data analysis. The findings highlight the importance of being *Valued for My Lived Experience Expertise*, which emerged as a central theme. The two primary themes were *Communication* and *Engagement*. *Communication* entailed students demonstrating *Active Listening* and *Attributes* and *Engagement* was seen through *Actions and Behaviours* and *Rapport*. The findings support the growing body of evidence highlighting the substantial impact of Experts by Experience on mental health nursing education. Integrating Experts by Experience into mental health nursing education enhances communication skills by improving students' understanding of mental illness directly from those who experience it. These changes are essential for advancing nursing education and improving mental health services.

## 1 Introduction

Mental health is recognised by the World Health Organization (2022) as a fundamental human right and a vital component of overall well‐being. Every individual is entitled to the highest standard of mental health care, yet the provision of such care in Australia remains inadequate (National Mental Health Commission 2023). Stigma and discrimination within the healthcare system, particularly in communication practices, exacerbate these issues. Individuals often report feelings of shame, devaluation and dehumanisation when interacting with healthcare professionals, which not only deters them from seeking or continuing care but also contributes to the growing prevalence of mental health challenges across the country (Knaak et al. 2015; Slade et al. 2014). Recent data indicate that nearly 1 (18.4%) in 5 Australians experienced high or very high levels of psychological distress in 2020, up from 13% in 2014 (Australian Bureau of Statistics 2022). This trend underscores the urgent need for systemic changes, particularly in how mental health care is communicated and delivered.

The Australian mental health system continues to struggle with significant stigma and discrimination, often perpetuated through ineffective communication practices within clinical settings (Mental Health Coordinating Council [MHCC] 2022). These communication barriers reinforce harmful stereotypes, create obstacles to care and strain therapeutic relationships, ultimately hindering personal recovery (Gwarjanski and Parrott 2017). Despite efforts to reduce stigma through awareness campaigns, advocacy work and educational initiatives, the entrenched institutional biases persist that slow the transition to a recovery‐focused system (Gee, McGarty, and Banfield 2016). The inadequacy of current mental health placements for nursing students further limits their ability to learn and practice communication skills in mental health settings effectively. Addressing these challenges is critical, and it is increasingly recognised that training nursing students to understand and combat stigma, particularly through improved communication, is essential. As the largest health profession, nurses are central to national health outcomes, and thus, investing in their education, especially in the context of mental health care, is vital for addressing Australia's urgent mental health needs (Morgan, Wright, and Reavley 2021).

Clinical placements, also known as work‐integrated learning or service learning, are pivotal in shaping nursing students' understanding of mental health care and communication (Lim et al. 2020). Programmes that immerse students in experiences with individuals who have lived experience of mental illness, such as Recovery Camp (RC), have been shown to reduce stigma and foster more positive attitudes (Moxham et al. 2016, 2017; Patterson et al. 2016, 2018; Morgan, Wright, and Reavley 2021). Recovery‐Oriented Care (ROC), which acknowledges individuals as the experts in their lives, supporting their journey of purpose, hope, connection, empowerment, meaning and fulfilment, is central to these placements (Leamy et al. 2011). ROC aims to foster mutual respect, support self‐determination, encourage goal setting, utilise strength‐based approaches and promote joint decision making (Morgan, Wright, and Reavley 2021). Such placements have demonstrated significant success in reshaping students' perceptions of mental illness and reducing stigma, making them a powerful tool in mental health nursing education (Foster et al. 2019). Strengthening these skills and understandings is beneficial and essential for improving the quality of care provided to Australians and building a more compassionate and effective mental health workforce, where respectful, person‐centred care underpins all therapeutic engagements (MHCC 2022).

### 1.1 Aim/Question

This study aimed to explore how individuals with lived experience of mental illness experience nursing students' communication during their participation in the mental health nursing clinical placement, RC.

## 2 Methods

Phenomenology is a methodology that aims to understand the conscious human experience, exploring how individuals perceive, interpret and attribute meaning to their world. This study used phenomenology to offer a nuanced understanding of how people with mental illness view students' communication in the RC clinical placement environment.

### 2.1 Study Setting

The study setting was RC in New South Wales, Australia. RC is a unique, recovery‐oriented clinical placement for nursing students and psychosocial intervention for people with mental illness. Participants engage in an immersive 4‐night, 5‐day programme promoting collaborative learning and shared experiences. Structured therapeutic recreation activities create a supportive environment, cultivating the space for meaningful discussions on mental health (Patterson et al. 2018). RC provides an ideal setting for exploring the research question through interactive student–Experts by Experience (EBE) interactions.

### 2.2 Sampling

Purposeful sampling ensured the selection of participants best positioned to provide detailed insights and enrich research findings (Polit and Beck 2017). The purposive study population ensured that the participant's knowledge and experience were relevant to the research question. According to Polit and Beck (2017), this is a common recruitment practice aimed at understanding a specific phenomenon or experience.

### 2.3 Inclusion Criteria, Study Participants and Recruitment

The inclusion criteria were people with lived experience of mental illness who had communicated with nursing students during RC, were over 18 years and had conversational English.

After institutional approval (2019/ETH03767), recruitment occurred after the May 2023 RC. All EBE received an email with an attached research brochure and the research team's contact details. Participants who expressed interest received a consent form and participant information sheet before scheduling interviews. Five participants (*n* = 5) took part in the study.

Throughout the study, the term ‘Experts by Experience’ refers to the participants, all of whom have first‐hand experience living with mental illness.

### 2.4 Research Rigour

Rigour and validity instil trust between the researcher, their findings and their readers (Thomas and Magilvy 2011). Dependability was maintained by keeping detailed records of the research process, including how data were collected and analysed. Confirmability was strengthened through ongoing reflection to minimise potential biases. These steps helped ensure the findings accurately reflect the participants' experiences and perspectives.

### 2.5 Data Collection

The first author completed individual, semi‐structured interviews, allowing for guided questions and the flexibility to explore participants' subjective experiences. Based on accessibility and convenience, participants could choose between face‐to‐face or videoconferencing formats. All participants chose videoconferencing via Zoom. Each interview was audio‐recorded and transcribed verbatim. The interviews ranged from 33 to 65 min, with an average duration of 48 min.

Data saturation was achieved using the constant comparison method. Key themes began to repeat within the first five interviews. This repetition indicated that no new significant information was emerging, which was appropriate given the project's time constraints.

A grand tour question commenced the interview, followed by focused inquiries to gather diverse perspectives and experiences on nursing student communication. Semi‐structured interviews allowed adherence to the research framework while exploring subjective responses (Kallio et al. 2016), facilitating deeper exploration of participant responses (DeJonckheere and Vaughn 2019). The semi‐structured questions were carefully constructed based on a thorough literature review. They were constructed with guidance from three academic supervisors with extensive knowledge and experience with semi‐structured questions of this nature.

### 2.6 Data Analysis

The study employed van Kaam's psychophenomenological model (PPM) for data analysis. PPM aligns with phenomenology, directly informing the research aim (Sumskis, Moxham, and Caputi 2017). It involves a 12‐step process, divided into four main sections (see Table 1), that guides the analysis to extract a meaningful understanding that aligns with the research aim.

**TABLE 1:** van Kaam's PPM 12‐step process of analysis.

Steps 1–8: Analysis
Step 9: Translation
Step 10: Transposition
Steps 11 and 12: Phenomenological reflection

The research team collected and de‐identified data and then independently reviewed transcripts to identify structural elements and themes specific to this study. Transcripts were re‐reviewed, repetitive statements were removed and themes were compared to the research objectives for relevance. Steps 1–8 were repeated to ensure the analysis accurately reflected participants' lived experiences, adhering to psychophenomenological situational reflection. No translation was needed in Step 9, preserving participants' voices in their original form. In Step 10, supervisors discussed findings to ensure alignment with the research aim. Step 11 involved comprehensive discussions among the research team to finalise the thematic synthesis. In Step 12, limitations were considered to acknowledge potential constraints. This systematic approach ensured a thorough analysis identifying key themes and meanings relevant to the study's objectives.

## 3 Results

**FIGURE 1:**
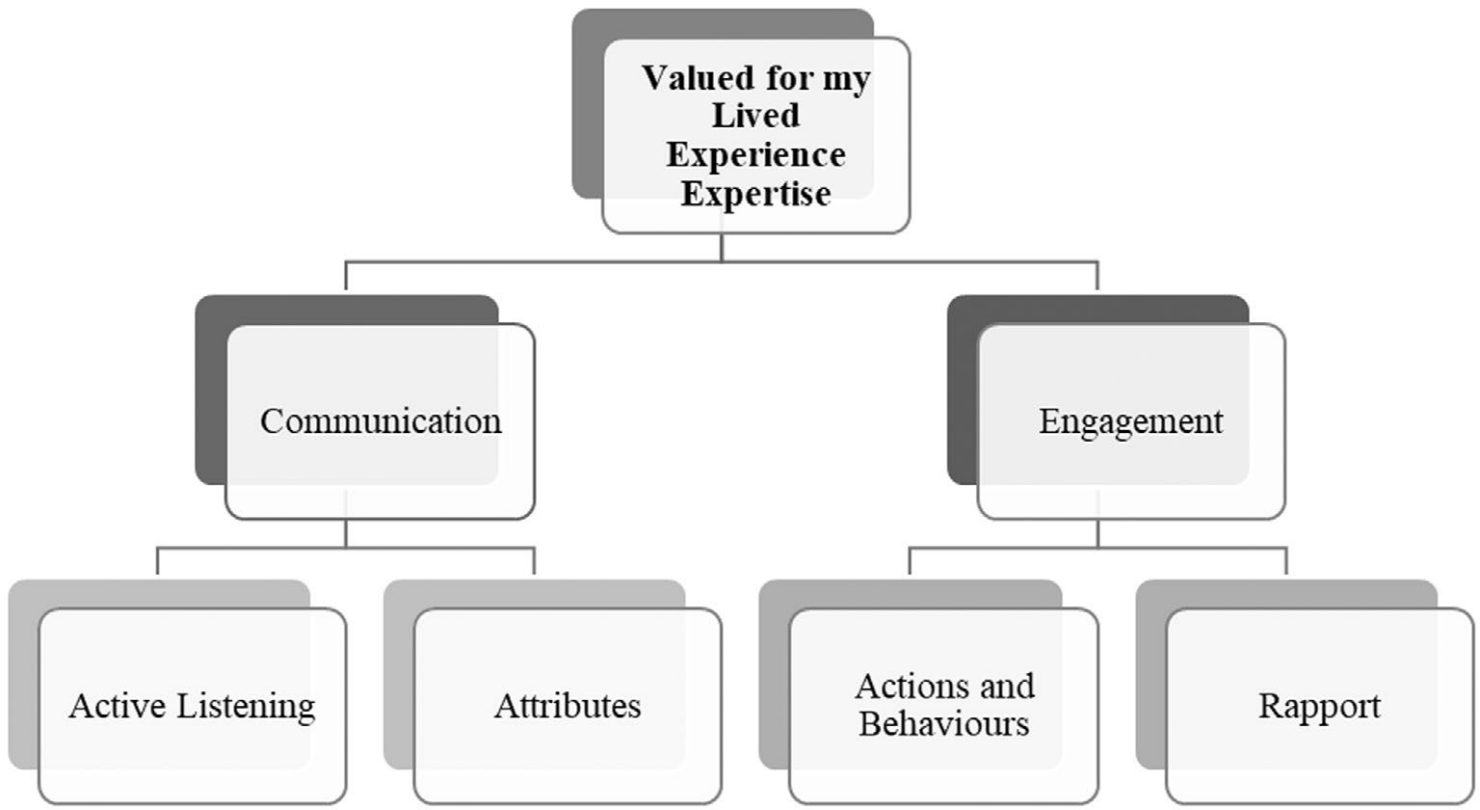
Essence of meaning and subsequent themes that emerged from the data.

Analysis of the data revealed four sub‐themes (*Active Listening*, *Attributes*, *Actions and Behaviours* and *Rapport*), two themes (*Communication* and *Engagement*) and the essence of meaning (*Valued for My Lived Experience Expertise*). The findings have been illustrated in Figure 1.

Development of the essence of meaning, *Valued for My Lived Experience Expertise*, was supported by the two themes: *Communication* and *Engagement*. These themes are underpinned by sub‐themes. *Communication* represents the collation of sub‐themes *Active Listening* and *Attributes*. The theme illustrated ways in which nursing students conveyed respect and interest in the participants' lived experiences. The related sub‐themes demonstrated that effective communication practices, such as empathetic listening and the display of positive attributes, were crucial in making participants feel valued. Similarly, the theme of *Engagement* represents the collation of sub‐themes *Actions and Behaviours* and *Rapport*. The theme *Engagement* relates to how students' proactive involvement and relationship‐building efforts contributed to the participants' perception of being respected and valued.

Together, the themes and sub‐themes reinforce the overarching essence of meaning illustrating the multifaceted ways nursing students' communication and engagement fostered a sense of recognition and appreciation for the participants' expertise.

### 3.1 Communication

The theme of *Communication* was developed from participants' experiences, showing that nursing students' ‘soft’ skills were identified as the most important element of their nursing practice. Participants consistently emphasised that elements of person‐centred communication were essential in establishing a relationship. This, in turn, allowed students to learn more effectively from EBE's lived experience expertise. For example, participant P stated that students could adjust their preconceived ideas about mental illness and improve their nursing abilities through these interactions. P expressed that these communications could ‘Change people's hearts about what mental health is and take a bunch of stigmas off and get some really good quality people in mental health nursing’.

Participants' narratives further clarified that effective verbal and non‐verbal communication significantly fostered trust and understanding. Michael's comment, ‘You can't help a client in a hospital or expert without good communication’, underscores this sentiment. This quote highlights the critical role that communication plays in establishing therapeutic relationships.

*Communication* included the sub‐themes of *Active Listening and Attributes*.

#### 3.1.1 Active Listening

Participants highlighted *Active Listening* as a critical aspect of communicating with nursing students. They described how *Active Listening* enabled genuine connections and promoted understanding and validation. For example, particiant VS noted, ‘If they are willing to sit there and listen to what I need to say and learn from it, then by all means, I'm happy to trust them with what I need to say’. This illustrates how participants interpreted *Active Listening* as a sign of respect and acknowledgement, which fostered trust.

Most participants felt that they experienced *Active Listening* from students, evidenced by empathetic prompts and acknowledgements that deepened conversations. VS expressed this sentiment by saying, ‘They actually listened, and they took it all in’. Similarly, Mate's statement, ‘They were listening’, reinforced the importance of being heard.

These examples demonstrate that participants perceived active listening as an essential communication strategy that allowed students to connect with them on a deeper level. Michael emphasised this by noting that students used open‐ended questions to get ‘people talking’ and prompts that enabled participants ‘to open up with students’. P echoed this sentiment, stating that they would ‘just keep on talking if given prompts’. Michael also highlighted the significance of the students' speech, stating that their ‘tone and volume is good’. Similarly, Mate pointed out the importance of voice and tone, noting that students had ‘a friendly tone of voice’. These insights accentuate the importance of verbal communication as a key component of effective interaction, which is why it emerged as a central theme in this study.

Non‐verbal communication was also crucial, with most participants expressing their ability to interpret nursing students' body language. VS explained, ‘I can tell a lot in a conversation with what's happening with body language’, and Mate added, ‘When someone is looking you in the eye, you know they're listening’. These observations indicate that body language significantly impacted participants' perceptions of students' communication and their willingness to engage.

Overall, *Active Listening* made participants feel that their life experiences were significant, valuable and respected. Participant P remarked, ‘You can just see it in their face. You can see that they're actually intrigued by who you are and what you've been through’. This connection fostered a sense of validation and genuine intent from the students.

#### 3.1.2 Attributes

In the context of *Communication*, participants observed positive *Attributes* in nursing students and described how these attributes made them feel. Participants frequently mentioned feeling cared for and supported by students, which enhanced their communication experiences. Rachel's statement, ‘Wow, we are just bathed in an environment of love and care. They have such big hearts and care’, encapsulates this sentiment.

The impact of encountering student positivity was so profound that participants questioned whether this resulted from their training or a natural trait. Michael wondered, ‘Are students meant to go overboard on love, or does it just come naturally?’ Participants appreciated students showing an interest in their stories, viewing this curiosity as a critical *Attribute* of students showing a desire to learn, explore and understand their experiences.

Participants identified open‐mindedness, inquisitiveness and a willingness to learn as crucial *Attributes* that positively influenced their interactions with students. P expressed this by saying, ‘You can pretty much tell the inquisitive ones that want to know that are interested in mental health’. Rachael echoed this with, ‘I got so much curiosity’. VS supported this sentiment: ‘Consistency of the nurses wanting to learn helped break down the wall a little bit more’, creating a space for therapeutic relationships to develop. Participants recognised these attributes, such as curiosity, empathy, kindness, compassion and respect, as essential in breaking down barriers and fostering more profound, more meaningful interactions. P highlighted this by noting that students were ‘open to learn about the expert's life’ and ‘how they cope daily’. These qualities were appreciated by the participants and contributed to a deeper exploration of their lived experiences. This is exemplified by Michael's account of a student saying, ‘Let's go outside and talk’, which led to a meaningful conversation about his life and illness. Such interactions underscore the powerful impact that these positive attributes have in creating a compassionate and engaging learning environment.

### 3.2 Engagement

The theme of Engagement represents how nursing students were perceived to interact meaningfully with EBE during the clinical placement. Engagement was characterised by *Actions and Behaviours* and *Rapport*, emphasising the importance of proactive involvement and relationship building as critical forms of nursing student communication.

#### 3.2.1 Actions and Behaviours

All participants believed that students' *Actions and Behaviours* demonstrated positive intent and were foundational in establishing a therapeutic relationship. Participants often recounted how students' inclusivity and kindness made them feel safe and valued. Rachael described this by saying, ‘Instantly I felt safe’. Michael shared a similar experience, noting, ‘I have one buddy in particular, who sat down next to me on the first day’, and Mate added, ‘They [students] just come sit down. Like, “Hey. Hey, what are you doing there?”’

These actions were not routine interactions, but were seen as genuine attempts to engage and build relationships. P shared how a simple game turned into an opportunity for meaningful conversation about living with bipolar disorder. This engagement created a safe space for discussing mental illness more openly, which P found transformative: ‘They kinda just stopped the hacky sack thing and asked, so, you know, “what's it like to live with bipolar or schizophrenia?”’

Participant Mate recounted a similar sentiment during a basketball game, where the *Actions and Behaviours* of the students clearly demonstrated their genuine intent to engage with EBE. Mate shared, ‘We were playing basketball till one in the morning … you never met before, and, still though, they were mixing with you like they have known you ten years’. This example illustrates how simple, shared activities created a sense of camaraderie and connection, making a lasting impression on participants.

Similarly, Michael reflected on a seemingly small act of kindness when a student made him a cup of tea, which profoundly impacted his experience. Michael explained, ‘The sense of providing for me, and it's an affectionate thing, and it's a nice thing’. This gesture communicated respect and sparked deeper conversations, nurturing an environment conducive to enriched interactions. Michael noted how these small acts influenced his willingness to engage further, stating, ‘I'm more likely to talk to them … ask some questions. They might make them more willing to talk to me and ask me questions’.

Participants recognised and valued these compassionate behaviours and supportive actions, significantly impacting their experiences and the relationships they built with students. Despite the brief duration of the clinical placement, participants felt that the proactive engagement quickly helped establish meaningful connections. P noted, ‘You might have a few of the students offering help with the survey on the first day. And so, sort of the ice is broken’. Michael supported this by saying, ‘Was only over a week, but they helped, and they cared, and they were friendly’.

Rachael also shared a moment during an activity at RC, where her initial hesitation due to fear of judgement was quickly alleviated as ‘everyone was cheering me as I walked’. This collective support from the students created an environment of acceptance and safety, allowing Rachael to feel more comfortable and supported through the student's *Actions and Behaviours*.

#### 3.2.2 Rapport

Building Rapport was not spontaneous but was the result of time, effort and consistent engagement. VS explained, ‘It honestly just starts like a small conversation. We just started talking about hobbies and things like that. I find that incredible that they are willing to get to know you a little bit first, and then once they've gotten to know you a little bit more, they can ask more difficult questions about your mental health problems’. This illustrates how Rapport developed gradually, starting with casual conversations and evolving into more meaningful connections.

Participants highlighted that *Rapport* was crucial in overcoming stigma and humanising their interactions with nursing students. P shared how students, after investing time and learning about his life, confronted their preconceived biases towards mental illness and told him, ‘You're not as scary as we once thought maybe you were’. Students were seen to have removed the ‘blinders and shutters’ they held towards EBE.

While most experiences with *Rapport* were positive, some participants did encounter negative experiences that hindered relationship building. VS described how a few students were dismissive, saying, ‘A couple of the nursing students were like, OK, yep, cool, we can see where we've gone wrong, but a couple of student nurses that just kind of shrugged it off and went well, you'll get over it’. Similarly, Mate discussed how a lack of engagement from students negatively impacted his perception: ‘You don't have the time for me, I don't have the time for you’.

**FIGURE 2:**
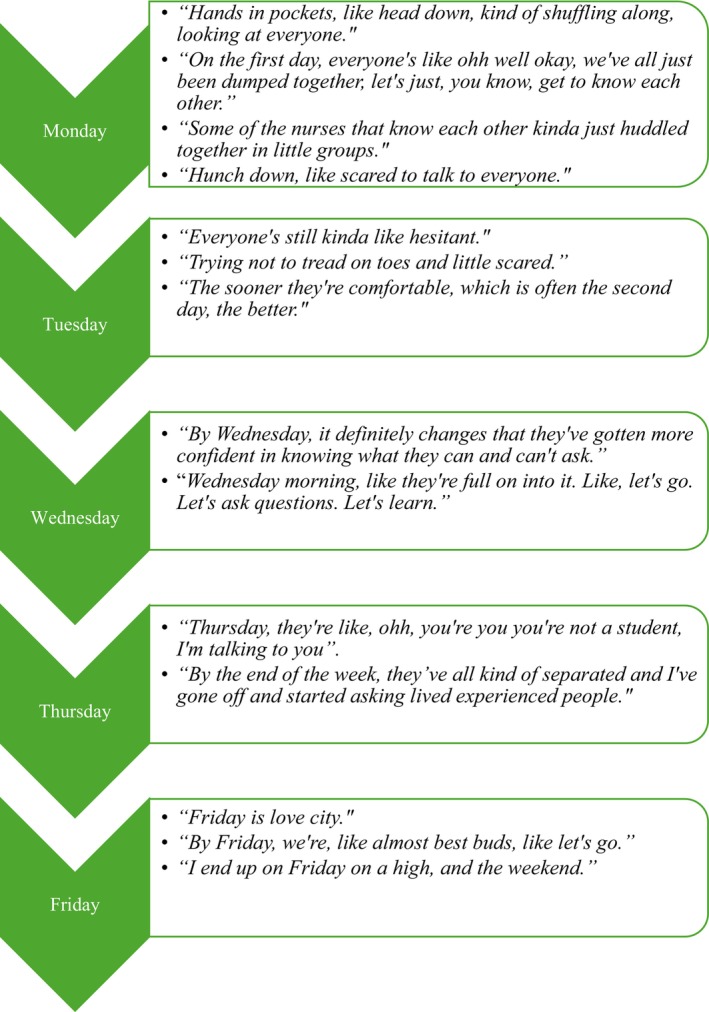
The building of rapport over time.

Despite these challenges, most participants felt that *Rapport* developed significantly over the 5‐day experience, with clear improvements in nursing students' communication as the week progressed. VS observed this growth positively, saying, ‘It's really nice just to see them grow a little bit from day one to day five’. Figure 2 shows participant views on the changes in *Communication* and *Rapport* as the week progressed.

### 3.3 Essence of Meaning: Valued for My Lived Experience Expertise

The essence of meaning from this study centres around the perception of EBE, who felt that through their interactions with students, their lived experience and expertise were truly acknowledged and valued. Participants expressed that being recognised as ‘experts’ by students was deeply impactful. The recognition affirmed their knowledge and experiences in a way that contrasted with their often disempowering interactions within the broader healthcare system. As Rachael put it, ‘They [students] call us the experts, that blew me away. That respect and honouring that we know about living with mental illness more than any doctors and specialists … I am loved, accepted and valued for who I am’.

This sense of being valued also instilled a deep sense of pride and purpose among participants. VS expressed, ‘I am taking part in something so big. Being able to teach a newer generation of nurses about struggles we have had to face, it's absolutely incredible. It makes me feel a little bit proud’.

This feeling of contributing meaningfully had a significant impact on participants' self‐perception. P described this effect, saying, ‘It gives very clear self‐worth. That my life is genuinely a useful tool for others to learn. And I think that is very valuable’. P also shared that students expressed their gratitude and appreciation, telling him, ‘It's completely changed my outlook on what mental health is. Thanks for being so open about your life and your journey. I'm not going to forget this’.

The importance of being valued and respected for their expertise was a recurring theme among participants. EBE recounted feeling pride and a sense of contribution, which were closely tied to their identities as educators and contributors to a significant cause. P highlighted this by stating, ‘I want to be valued, and I want my information to be valuable’. VS echoed this sentiment: ‘It makes me feel that there's hope yet for the system’.

Participants consistently felt that their lived experiences were valuable tools for students to learn about mental illness and that their contributions were respected and appreciated. They believed that their role as educators could have a meaningful impact on improving nursing students' understanding of mental illness, ultimately contributing to better mental health care within the nursing workforce.

## 4 Discussion

This study presents novel insights into the role of communication and engagement in nursing education, specifically within the context of RC. Unlike traditional placements, RC is an immersive environment that allows for interactions between nursing students and EBE. The environment can lead to a significant reduction in stigma and a deeper understanding of mental health recovery. The importance of lived experience in education has been acknowledged in previous research; however, this study is unique in its focus on communication as a core element. The findings reveal that through exposure, *Communication* and *Engagement* improved, which is not only a necessary nursing skill, but also essential in challenging and reshaping their perceptions of mental illness. These contributions offer a new perspective on the value of EBE‐centred education and suggest innovative strategies for improving nursing curricula, which will be further explored in the subsequent sections of this discussion.

### 4.1 Communication

This study highlights the critical role of *Communication* in shaping the interactions between nursing students and EBE. The findings indicate that effective communication, characterised by *Active Listening* and underpinned by a student's *Attributes*, fosters respectful and meaningful connections. This aligns with existing research, such as Vogel, Meyer, and Harendza (2018), which emphasises *Active Listening* as a critical factor in perceived respect. Blanch‐Hartigan et al. (2018) also support this by demonstrating that open body language, eye contact and receptive non‐verbal cues enhance the listener's authenticity. Wanko‐Keutchafo, Kerr, and Jarvis (2020) also argue that non‐verbal cues play a pivotal role in shaping communication perceptions, again supporting the study's findings. Participants' positive reception of these cues mirrors Shih et al.'s (2023) findings, where service users in healthcare settings reported higher satisfaction when they perceived staff as actively listening. These studies collectively suggest that effective communication involves non‐verbal behaviours that convey compassion, empathy and respect (Quinn 2016). Broader literature supports the findings of this study, showing that nursing students are meeting elements of effective communication, which is essential to ROC by nursing students.

This study explores communication dynamics and reveals the significance of personal attributes such as empathy, compassion and respect in influencing how EBE perceive communication. While these attributes are fundamental to nursing practice (International Council of Nurses 2023), their consistent application in practice remains challenging. Studies such as Taylor, Thomas‐Gregory, and Hofmeyer (2020) highlight deficiencies in care delivery linked to perceived unprofessional attitudes among staff, while Gras et al. (2015) identified poor care outcomes associated with staff attitudes. Another study found that students had negative attitudes in the category of stereotypes when engaging in mental health clinical placements (Lim et al. 2020). Furthermore, additional environmental factors that have been known to affect communication are well documented, such as time constraints, understaffing and excessive workloads (Norouzinia et al. 2015). These challenges are notably less pronounced in the immersive setting of RC, where the clinical placement's ongoing nature allows for deeper, more meaningful interactions. This highlights the importance of creating environments conducive to effective communication, particularly in the context of education. This shift suggests that immersive experiences, such as those provided by RC, have the potential to challenge and change stigmatising attitudes through direct interaction with EBE. Research on RC supports this finding, with studies by Moxham et al. (2016) and Picton et al. (2018) highlighting the programme's success in creating a transformative learning environment where students can re‐evaluate their assumptions about mental illness and develop a more empathetic approach to care.

The unique environment of RC, which facilitates close interaction between students and EBE, plays a crucial role in fostering attitudinal changes. Goman et al. (2020) and Picton et al. (2018) have detailed how RC helps students address underlying stigmatising attitudes, leading to a more holistic understanding of mental health. Moxham et al. (2016) found that students who attend RC are less stigmatising compared to their peers who attend traditional placements. The immersive nature of RC contrasts with traditional placements, where students may not experience the same level of engagement or opportunity for reflection.

This study adds to the growing body of evidence that effective communication in mental health nursing is not only a matter of verbal exchange, but also involves the demonstration of key personal attributes and the appropriate use of non‐verbal cues. The findings reinforce the importance of immersive, EBE‐centred clinical placements in overcoming communication barriers and reducing stigma, impacting the overall quality of care in mental health nursing.

### 4.2 Engagement

Participants in this study consistently reported feeling welcomed and valued when nursing students exhibited certain behaviours. *Actions and Behaviours* are crucial in shaping how *Communication* is interpreted, with Ekpenyong et al. (2021) noting that these behaviours, even when unrelated to direct nursing tasks, significantly influence patients' perceptions. Alikari et al. (2022) further emphasise this when finding that patients prioritise a nurse's behaviours over their clinical knowledge. This study's findings align with these observations, as participants noted that students engaged in inclusive, kind and respectful behaviours. This is particularly important given that research by Akansel et al. (2021) and Mårtensson et al. (2023) indicate that nurses often struggle to consistently apply these behaviours in practice despite their essential role in nursing. Nursing education often prioritises clinical tasks over interpersonal skills, focusing more on ‘doing’ rather than ‘being’ (Taylor, Thomas‐Gregory, and Hofmeyer 2020). Zamanzadeh et al. (2014) found that nursing students tend to prioritise theoretical knowledge and practical skills over building trust and human connections. Similarly, Akansel et al. (2021) observed that students often place more importance on physical nursing skills than on verbal and non‐verbal communication, a focus that Glantz, Örmon, and Sandström (2019) argue overshadows human interaction.

Educational interventions, such as clinical placements, play a pivotal role in shaping nursing students' behaviours (Karami, Farokhzadian, and Foroughameri 2017; Goy et al. 2021). Understanding the interconnectedness of *Actions and Behaviours* and their perception as key components of effective communication is vital to the nursing curriculum (Culha and Acaroglu 2019). In this study, participants observed kind and inclusive behaviours among the student nurses, with many noting improvements and growth in these behaviours throughout their time at RC. This indicates that immersive, EBE‐centred education, such as that provided at RC, facilitates the evolution and development of positive actions and behaviours in nursing students, ultimately enhancing their capacity for compassionate care. The findings of this study support the evidence suggesting that EBE‐involved education is an effective recovery‐based education tool and a recommended way forward in nursing curriculum (Goh et al. 2021).

### 4.3 Essence of Meaning

The findings from this study emphasise the benefits of EBE participation in education. Participants felt valued for their lived experience and expertise. Numerous studies (Goman et al. 2020; Patterson et al. 2016; Picton et al. 2018) highlight the significance of EBE‐involved undergraduate nursing education and its positive impact on students. Observations by Bocking et al. (2019), Ridley, Martin, and Mahboub (2016) and Moxham et al. (2016) indicate reduced stigma in students and a shift in their perceptions of mental illness when exposed to EBE‐centred education. In addition, EBE felt valued and had a new sense of self‐discovery through their role as educators, a finding that is continuing to be explored in literature (Yousiph et al. 2023

However, despite its benefits, EBE‐integrated education remains uncommon (Happell et al. 2016), and its value and implementation are not widely reflected in nursing education guidelines or curricula (Happell et al. 2018). Concerns regarding consumer qualifications, funding challenges and a lack of guidance on fostering this partnership persist (Fraser et al. 2017). These considerations show the necessity for further research into EBE‐centred education. Despite the conflicting evidence in broader research into EBE‐integrated education, this study supports its place in education. All participants unequivocally recognised their profound contribution to students, highlighting their indispensable value in the educational setting.

## 5 Limitations

The constrained sample size limits representative diversity. Focusing on five participants allowed for an in‐depth analysis, but restricted generalisability. Additionally, the study's time frame, limited to a university‐structured honours year, offers a snapshot rather than comprehensive insight. Longitudinal studies or larger samples could offer different perspectives.

## 6 Conclusion

The study suggests that to prepare nursing students for the complexities of mental health care, there must be a stronger emphasis on incorporating EBE into teaching. Adopting a more immersive recovery‐based education across all nursing programmes should be considered essential. While the importance of recovery‐oriented education is widely acknowledged, this research highlights that it must be complemented by hands‐on learning opportunities like those provided at RC. Such placements can address existing gaps in nursing education, equipping students to work confidently and competently in mental health settings.

## 7 Relevance for Clinical Practice

Integrating EBE into mental health nursing education is crucial for enhancing communication skills and fostering a deeper understanding of mental illness within the emerging nursing workforce. The research findings emphasise the significant role that EBE plays in reducing stigma, humanising mental illness and improving the overall capability of the nursing workforce. Additionally, the study underscores the positive impact on EBE who experience a sense of value and recognition for their lived experience expertise. Understanding these dynamics is essential for refining the nursing education model in Australia, ultimately enriching students' learning experiences and improving mental health services by integrating lived experience.

## Ethics Statement

Ethical approval was obtained from the relevant institutional ethical board (approval no: 2019/ETH03767—General Amendment 131540).

## Conflicts of Interest

Recovery Camp is a research and social impact programme that has been supported by the University of Wollongong to explore its potential as a social enterprise. Christopher Patterson and Lorna Moxham are directors of Recovery Camp Pty. Ltd.

## Data Availability

The data that support the findings of this study are available on request from the corresponding author. The data are not publicly available due to privacy or ethical restrictions.
